# A Qualitative Study to Understand the Barriers and Facilitators in Smoking Cessation Practices Among Oncology Health Care Practitioners in One Health System

**DOI:** 10.1093/ntr/ntae185

**Published:** 2024-07-22

**Authors:** Kate Frazer, Nancy Bhardwaj, Patricia Fox, Ailsa Lyons, Shiraz Syed, Vikram Niranjan, Amanda McCann, Catherine Kelly, Sinead Brennan, Donal Brennan, James Geraghty, Michael P Keane, Patricia Fitzpatrick

**Affiliations:** School of Nursing, Midwifery and Health Systems, University College Dublin, Belfield, Dublin, Ireland; School of Public Health, Physiotherapy and Sports Science, University College Dublin, Belfield, Dublin, Ireland; School of Nursing, Midwifery and Health Systems, University College Dublin, Belfield, Dublin, Ireland; Department of Preventive Medicine and Health Promotion, St. Vincent’s University Hospital, Elm Park, Dublin, Ireland; Department of Preventive Medicine and Health Promotion, St. Vincent’s University Hospital, Elm Park, Dublin, Ireland; School of Public Health, Physiotherapy and Sports Science, University College Dublin, Belfield, Dublin, Ireland; Conway Institute of Biomolecular and Biomedical Research, University College Dublin, Belfield, Dublin, Ireland; School of Medicine, University College Dublin, Belfield, Dublin, Ireland; Mater Misericordiae University Hospital, Dublin, Ireland; St Luke’s Radiation Oncology Network, Rathgar, Dublin, Ireland; School of Medicine, University College Dublin, Belfield, Dublin, Ireland; Mater Misericordiae University Hospital, Dublin, Ireland; School of Medicine, University College Dublin, Belfield, Dublin, Ireland; St Vincent’s University Hospital Group, Elm Park, Dublin, Ireland; School of Medicine, University College Dublin, Belfield, Dublin, Ireland; St Vincent’s University Hospital Group, Elm Park, Dublin, Ireland; School of Public Health, Physiotherapy and Sports Science, University College Dublin, Belfield, Dublin, Ireland; Department of Preventive Medicine and Health Promotion, St. Vincent’s University Hospital, Elm Park, Dublin, Ireland

## Abstract

**Introduction:**

Despite the benefits of quitting smoking for those who have cancer, including improved health outcomes and reduced therapeutic toxicities, it is unclear how many people are supported in quit attempts during this time. Variations in the availability and provision of smoking cessation (SC) services are reported, with little understanding of the challenges and solutions. This codesigned study aimed to understand the perspectives of health care professionals (HCPs) working in oncology settings to engage in SC practices and identify recommendations for developing a SC pathway.

**Aims and Methods:**

This was a qualitative study. Eighteen HCPs participated in semi-structured interviews from July 2021 to May 2022. We used thematic analysis approaches to code data and present four themes and SC strategies at micro, meso, and organizational levels.

**Results:**

Four themes are reported specifically: (1) timing and knowledge, (2) building a relationship, (3) frequent asking with infrequent action, and (4) removing the barriers and tailoring the system. While HCPs discuss SC, there are variations in documentation and when conversations occur. Primarily, HCPs value the time to build therapeutic relationships with patients and thus may limit SC discussions in preference to treatment in clinical interactions. The role of structural barriers, including prescriptive authority for nurses, hinders active SC processes, as it is the lack of continuity and embedding of services supported by a clinical champion for SC.

**Conclusions:**

The study suggests reevaluating the status quo in SC service, highlighting service gaps, and suggesting opportunities at organizational levels to reduce structural barriers.

**Implications:**

Variations in SC services exist in designated cancer centers. The data from this study can be used to inform a real-time health systems approach for SC services in oncology settings. Developing tailored SC services and interventions that are patient-centered and informed by their experiences is required. The data in this study suggest developing specialist education and training to upskill HCPs for equitable engagement if we are to meet EU and Cancer Moonshot goals for cancer reduction.

## Introduction

Smoking is a leading cause of global premature mortality and morbidity.^1^ Despite reducing smoking rates in high-income countries, elevated prevalence rates continue in lower- and middle-income countries. Smoking is a principal cause of cancer, with more than 19 million new cancer cases diagnosed in 2020 worldwide and 10 million deaths reported.^2,3^ This is set to increase, with an expected 30 million new cancer cases diagnosed annually and 16 million deaths by 2040.^1,2^ The impact of continued smoking after a cancer diagnosis includes an increase in *all-cause* and *cancer-specific* mortality, more significant toxicity from therapeutic interventions, increased incidence of recurrence, and the risk of second malignancies.^4^ A 30%–40% reduction in the risk of dying is reported with quitting smoking at the time of cancer diagnosis.^4^

Evidence of variation in the availability and provision of smoking cessation (SC) services in oncology settings is reported.^5–9^ Few cancer patients engage with SC, many continue smoking, and some may not understand the link between continued smoking and increased risks. Moreover, health care professionals (HCPs) may ask about smoking history infrequently and have limited knowledge or time to engage despite articulating SC as a “*teachable moment.*”^5–7^ Inconsistencies continue in providing SC services, a finding reported a decade earlier in general hospitals in the UK,^6,9–11^ with variability in HCPs’ perceptions of the benefits of quitting for those who have cancer due to *fear of implying or exacerbating patients’ feelings of guilt.*^5^ In Ireland, the provision of SC services includes a national Quitline, availability of SC services, where available (includes individual and group supports), and access to free nicotine replacement therapy (NRT) (no payment required) in publicly funded hospitals and community settings. NRT can also be purchased directly in pharmacies in Ireland. Lyons et al.’s national audit of Irish publicly funded cancer centers confirms variability in SC services in four key areas including (1) not all patients are offered NRT, (2) an absence of a protocol guiding SC medication management in all cancer centers, (3) limited documentation of smoking status of patients, and (4) limited recording of HCPs’ compliance with the national Make Every Contact Count (MECC) training program (Brief Intervention Training).^11^ Fitzpatrick et al.’s review of inpatient data also confirms that smoking rates among cancer patients increased during 2014–2017, followed by a small reduction of 0.3% in 2018, while reporting the highest smoking prevalence among 50- to 59-year-old cancer patients (14%–15.3%) and for males during 2014–2018 among those with lung cancer (24%–27%).^8^ Thus, it is imperative to consider alternatives if we are to meet the EU targets for cancer control^12^ and current US Cancer Moonshot goals.^13^

Creating an SC pathway requires considerable insight from all involved in developing a complex intervention … *taking into account how it interacts with the context in which it is implemented and how the evidence can be used to support real-world decision-making*.^14^^(p. 1)^

## Materials and Methods

### Study Design

An exploratory qualitative study design was used, with reporting guided by Standards for Reporting Qualitative Research (SRQR).^15^

### Aim

The study aimed to explore the SC practices, experiences, and opinions of oncology HCPs working in three of the nine public specialist cancer settings in Ireland and to seek recommendations to develop an SC pathway.

The research question guiding the study was:

What are the SC practices, experiences, and opinions of oncology healthcare professionals, and their recommendations for developing a specialist SC pathway?

### Recruitment and Sampling Strategy

HCPs caring for adults diagnosed with lung, breast, cervical, head and neck, and other cancers were recruited from three national centers in Ireland (there are eight specialist centers and one radiation oncology network in Ireland) providing publicly available services. Recruitment and data collection for the study commenced in July 2021 for 10 months (up to May 2022). Recruitment processes coincided with COVID-19 in 2020 and were adapted accordingly (delayed commencement of the study) as clinics were canceled and *in-person* hospital attendance ceased.

Purposive, maximum variation sampling^16^ was used to obtain a range of HCPs employed as front-line practitioners in oncology and radiotherapy settings (HCPs include registered nurses, medical doctors, surgeons, and oncologists, and allied HCPs include physiotherapists, dietitians, psychologists, and social workers) thus ensuring the inclusion of key informants and diverse practitioners who comprise multidisciplinary teams. Invitation letters and information sheets, including contact details of two study researchers for further information, were emailed and posted to nominated study gatekeepers (consultant oncologists and senior specialist nurses in the three selected centers), and they were asked to distribute the information within the multidisciplinary teams in each specialist center. If a practitioner was willing to participate, they signed consent forms collated by the study gatekeepers. Verbal consent was reaffirmed before the commencement of interviews, and agreement to record was obtained. Participants were informed that they could withdraw from the study at any time.

### Topic Guide

A topic guide was developed with specialist input from the research team and a study steering committee (SSC) that included three patients from a nationally representative network of people who previously had a cancer diagnosis, had a history of smoking, and were “*experts by experience*” (additional members included consultant oncologists, specialist in public health medicine, specialist practitioners in SC, nurses, and a dietician). Questions included were identified from published literature^9^ and designed to seek data on current SC practices, barriers and facilitators, training, expertise, and recommendations for developing specialist pathways. The topic guide was pilot-tested, and questions were modified, ensuring flow and sequencing and a short *pro forma* sought demographic data (Supplementary File 1).

### Data Collection

Eighteen semi-structured interviews were conducted by three study team members (NB, PFo, and SS) by phone or via virtual platforms, Zoom and Webex (with permission), and were digitally recorded. The duration of the interviews was 30–40 min. All participants were reassured that the data were confidential, reports would be anonymized, and data would be reported as group summaries, with no individual or cancer center identifiable.

### Ethical Considerations

Ethical approval was obtained from the Human Ethics Research Committees in three centers (RS21-021 Ethics/1/378/2236).

### Data Analysis

Data were typed verbatim by a professional transcription service. Identifying information was removed, and participants’ data were pseudo-anonymized, via an assigned code for identification. A thematic analysis approach was used “*identifying, analysing and reporting patterns (themes) within data*.”^17(p.57)^ Transcript data were migrated into Microsoft Excel worksheets for line-by-line review. Two research team members (NB and SS) completed the coding and identification of emergent themes for discussion with moderators. The coding process continued following discussion and agreement with moderators (PFi and KF), confirming four thematic areas anchored in the data using an inductive approach and not preassigned *a priori.* Data saturation was evident in the richness of data and using the definition when “*no new codes were determined during data collection and from data analysis.*”^18^ Themes were compared to determine where they converged, complemented, or diverged and were finalized following scrutiny and reflexivity by the experienced study team.^19^

### Trustworthiness

Trustworthiness relies on a study’s credibility, transferability, dependability, confirmability, and the researchers’ reflexivity.^20^ Coding and themes were developed based on the data content, and to safeguard against research bias, we kept reflexive notes and discussed interpretations of findings. Information on the recruitment process, participant characteristics, and data generation is reported to enhance transferability and dependability. To strengthen confirmability, we include quotations to substantiate our interpretations.^18^

## Results

### Participant Demographics and Smoking Cessation Practices

Participants from the three specialist centers comprised registered nurses (*n* = 8; 44.4%), clinical doctors (*n* = 7; 38.9%) (physicians, surgeons, and oncologists), and Allied HCPs* (*n* = 3; 16.7%) (Table 1). Less than a quarter had smoked previously (23.5%, 4 of 17), and a majority engaged in SC conversations (72.2%, *n* = 13), though fewer discussed e-cigarettes/vapes (44.4%, *n* = 8).

**Table 1:** . Demographic and Smoking Cessation Practice Characteristics of Participants Working in Public Hospitals

Participant HCPs	Professional group	Professional years of experience in specialism	History of smoking	Engages in smoking cessation conversation	Engages in a conversation about e-cigarettes/vaping
1	Doctor	15	None	No	No
2	Doctor	5	None	Sometimes	No
3	Doctor	10	Unknown	Yes	No
4	Nurse	4	None	Yes	No
5	Nurse	7	None	Yes	Yes
6	Allied HCP^*^	14	Previous	Yes	Yes
7	Allied HCP^*^	16	None	No	No
8	Nurse	15	Previous	Yes	Yes
9	Nurse	20	None	Yes	Yes
10	Allied HCP^*^	14	None	No	Yes
11	Nurse	13	None	Yes	Yes
12	Doctor	2	None	Yes	No
13	Nurse	17	Previous	Yes	No
14	Nurse	6 months	Previous	Yes	Yes
15	Doctor	4	None	No	No
16	Doctor	2	None	Not routinely	No
17	Nurse	3.5	None	Yes	Yes
18	Doctor	+20	None	Yes	No

^*^Allied health care professionals include health care professionals who are not nurses or medical doctors.

Doctor in this variable includes all medically employed practitioners working in the included centers as surgeons, physicians, or oncologists.

Nurses in this variable refer to all registered nurses in any of the included centers.

Due to ethical approval conditions, generic terms are used.

Four overarching themes were developed from the data, and quotes are presented below and in Supplementary File 2:

1. *Timing and knowledge*

This theme presents the experiences of practitioners in providing SC information and the impact of own knowledge base and perceptions on commencing a discussion.

1. *Building a relationship*

This theme focuses on practitioners’ prioritization and development of the therapeutic relationship in clinical encounters.

1. *Frequent asking with infrequent action*

This theme focuses on the process of brief intervention communication and the limited systems approach for referrals to SC services. HCPs were asking about smoking histories from patients, but it did not result in active actions and more of a passive system of communicating what patients had to initiate themselves.

1. *Removing the barriers and tailoring the system*

This theme focuses on the solutions that participants prioritized to develop future SC services.

#### Timing and Knowledge

The majority of participants were experienced HCPs working in cancer care (Table 1). Variations emerged in practices and interactions concerning the content of the advice and the timing of the SC conversations. Workload and competing conversations were identified as taking precedence, highlighting structural challenges of the health system. Additionally, personal challenges among participants are noted in discussing smoking and providing advice, including lack of time during all consultations due to workload in clinical settings, prioritizing time for competing priorities, and for others noting lack of knowledge of SC services in their organization. Participants routinely asked about smoking habits, mostly during initial consultation/at diagnosis (initial assessment) or before starting treatment/as part of treatment (presurgery/prechemotherapy/anticancer treatment/preradiotherapy) workup. It was acknowledged that information prompting the conversation could be based on written clinical medical notes and referral information received by oncologists, doctors, or nurses, as demonstrated below (and in Supplementary File 2):

… we include the information on smoking cessation and will bring up the topic again and will go through again the reasons why we would recommend them stopping and if they would be willing to do that, and what the supports available are. So we would go through it again with them after the consultation (HCP 11, Nurse 13 years’ experience).

Participants noted variability in clinical history assessments and the impact of their personal choice on whether to include questions on smoking. Questions on smoking were not routinely included for all patients and would be prompted by clinical questions or presenting symptoms, including (1) increased post-op morbidity, (2) a history of shortness of breath, (3) if patients take breaks to smoke outside during inpatient stays, or (4) specifically with patients diagnosed with head and neck or breast cancer. Consistent engagement in SC history as part of the clinical assessment was noted for patients diagnosed with lung cancer.

I would say probably about, to be honest with you, 80% of the time, not all of the time. I would say about 80% of the time I would ask if people smoke… (HCP 12, Doctor, 2 years’ experience).

While SC was a topic embedded in clinical assessment, for participants, personal decision-making diminished the importance of SC to secondary information for a minority, with professional judgment used to consider the appropriateness of the conversation. Time limitations were articulated, impacting clinical consultations. The systems challenge (a lack of time) is due to the organization of clinics, with finite duration and increasing number of consultations, which preempts restrictions in therapeutic engagement as described:

So I think, we kind of rush into clinic to get patients in quickly, to get them on treatment quickly but we don’t take a longer term view, generally, apart from giving them some fairly bland advice on specific radiation toxicities (HCP 1, Doctor, 15 years’ experience).

You have to time it, and you have to pick the correct patient. For some patients, if you ask that question, and they are smoking or continuing to smoke, it can be a barrier to being able to conduct a consultation. Or sometimes you know you can see in the notes that this has been discussed before and you can tell that they’re continuing to smoke. I would generally bring it up towards the end of the consultation rather than at the start, or in the middle, because if you bring it up too soon, sometimes the walls can go up and that means that your interaction for the rest of consultation around the rest of their health issues can be impacted. So yes, I do usually ask it, you know, probably not always (HCP 12, Doctor, 2 years’ experience).

Participants did not agree on the specific timing to commence a SC conversation. Specifically, their perceived difficulties and a lack of awareness of SC support influenced engagement with the process. Personal knowledge base, values, understanding, and experiences impacted the choice of whether to engage in SC conversations, as demonstrated in the following statements presenting “*the wrong time*” and the personal reflection.

Obviously, when someone is diagnosed with cancer that isn’t for them the right time to stop. Or they might try and they relapse and they go back smoking (HCP 11, Nurse, 13 years’ experience).

I would struggle to think that I have the time and I would struggle to be confident that I have the expertise, the knowledge, education, actually sees especially about replacement therapies and what products are available out there, so no disrespect, but I think it’s something that senior physicians are researching should highlight, and then I think somebody else should come in to give them the time and the knowledge to make informed decisions (HCP 3, Doctor, 10 years’ experience).

#### Building a Relationship

Participants valued the therapeutic relationship, which ensured a nonjudgmental approach in clinical interactions. Empathy and disclosure were pivotal—acknowledging the challenges for patients in losing control of life decisions at a time of significant personal upheaval and uncertainty. While all HCPs reported adopting an empathetic approach, broaching the topic of SC gently or casually to limit adding additional pressure on patients to quit and engaging using a direct approach style was also considered by participants who did not feel SC a taboo or sensitive topic.

I don’t see it as sensitive I said something that they have to consider and they have to. Look at delivering on that particular aspect of the treatment plan, because it could make a difference and different it could make a difference to the short term and long term outcomes (HCP 3, Doctor, 10 years’ experience).

I said, it’s very important to be very gentle with them and you’ve just met them, you’re forming a relationship, you don’t want to kind of be, you can’t be judgmental (HCP 9, Nurse, 20 years’ experience).

Knowing the person is an essential component of person-centered care and implicit in building relationships, using touchpoints for communication and interactions throughout the therapeutic relationship, encapsulating a lifecycle of diagnosis and survivorship—journeying beyond the initial consultation:

So I think it’s maybe just having a little bit of a rapport with them on building up a little bit of a relationship and then being able to say okay well look this is a part that we have to discuss at the end, it’s part of your care. And so I do think it’s having that a bit of a relationship with them and that they can be open and honest with you. Sometimes I think as a nurse, they may be are a little bit more open to talking to you about it, I think with a consultant they’re very much you know, obviously they may be a little bit intimidation (HCP 8, Nurse, 15 years’ experience).

Prioritizing the therapeutic relationship required trade-offs when considering the timing and content of SC conversations, as the well-being of patients was paramount, including not contributing to discussions that would increase patients’ feelings of guilt or stigma associated with smoking. The conversations were thus pragmatic and clinically focused, with information on the harms of smoking on treatment/radiotherapy toxicity/wound healing and recovery communicated when judged appropriate, as demonstrated below:

Like a bit nervous sometimes, like I said in terms of jeopardising the interaction. I know I spoke about the risks of asking the question but having a good rapport a non-judgmental rapport with the patient can be really helpful (HCP 12 Doctor, 2 years’ experience).

And it’s usually once they start treatment, we would just have a chat to them and just bring it up very casually ‘are you a smoker’ and just try and open up a conversation about. I think you have to be very, you know, you have to be very cautious taking it on as well, because obviously that they’re so stressed with everything that’s going on in their lives, so just the kind of brief interviews, brief questions ‘Are you smoker or have you ever considered giving up’ and that’s usually kind of once I’ve got to know them and seeing the maybe once or twice, when they are starting their treatment and then we’re seeing them regularly. So it’s easier to kind of I think start off gently with them and then just maybe give it a little bit more time once you develop a relationship with them (HCP 9, Nurse, 20 years’ experience).

#### Frequent Asking With Infrequent Action

While providing SC advice was a component of a professional role, active assistance in navigating the health care system, beyond sharing contact information or national quit line number, was less clear. Participants described disconnected SC services—detached from cancer services. Asking patients about their smoking status and connecting to the system varied. Participants described a system requiring patients or family members (if included in discussions) to communicate with publicly provided SC practitioners. Variability in conversations was influenced by a patient’s verbalized interest in quitting during clinical conversations. No active electronic system for referring to SC services exists:

I probably don’t offer formal referral to the smoking cessation service enough, I would say, and that’s because there’s a lot of information at that first consultation (HCP 2, Doctor, 5 years’ experience).

… if the patient tells me they are interested in stopping smoking. I don’t usually… I just ask are they interested and if they tell me they are interested in stopping smoking then I do make some suggestions (HCP 18, Doctor, +20 years’ experience).

As identified in Table 1, a majority of participants provided information on SC services (usually written leaflets, connecting patients to a hospital SC facilitator—where employed), providing information on the SC services if available in the hospital, national Quitline information, and prescribing NRT. It is clear that a systematic approach and embedding SC referrals within letters to General Practitioners (GPs) or referrals to specialist psychology services are not routine. Table 1 data also confirm that a minority of participants discussed e-cigarette use as part of a smoking assessment.

Sometimes I would direct them to the website *HSE Quit.ie*. But I probably wouldn’t normally talk about community smoking cessation or the GP... (HCP12, Doctor, 2 years’ experience).

I don’t routinely ask to be honest. (HCP 16, Doctor, 2 years’ experience).

#### Removing the Barriers and Tailoring the System

Structural barriers within the SC system included a lack of prescribing authority for nurses and knock-on delays in waiting for NRT prescriptions. Involving family members as part of an active engagement with SC discussions highlighted dilemmas and a balancing act of weighing the positives over the potential negatives due to unknown knowledge of smoking behaviors of family members.

We talk about the patches most of the time, and then I know there is the availability of gums, e-cigarettes I don’t usually advise, but the patch is the main thing, the patch and the gum usually (HCP 17, Nurse, 3.5 years’ experience).

I think it is very ad hoc, it is not consistent and there is not a whole lot of facilitation done being really honest. They don’t generally involve family members really; I don’t think they do and it is very ad hoc. So I suppose the facilitators that they would… No it is not really consistent, there is no point in saying it is, it is not really consistent (HCP 13, Nurse, 13 years’ experience).

Participants’ suggestions to improve the current system demonstrated empathy and compassion to overcome the structural barriers experienced by patients and the impact of the social determinants of health that innately compound why someone may be smoking. Being nonjudgmental was critical, acknowledging the stress and loss of control over self as experienced by patients when dealing with a cancer diagnosis, treatments, or relapse. Barriers to current SC services include lack of availability and organizational issues. It is evident that patients’ challenges—the need for personal capacity, literacy/communication issues, and capability to connect with unfamiliar facilities outside of cancer services—are understood by HCPs. What will help in considering a tailored SC pathway is demonstrated in the reflections below and presented in Table 2:

**Table 2:** Removing Barriers and Tailoring a Pathway

Patient focused solutions^1^		Hospital focused solutions^2^		Systems level focused solutions^3^	
*Tailored service*	Participant 1	*Structured pathway developed and communicated*	Participants 1, 2	*Social media used*	Participant 10
*Visible service and clear information*	Participant 1	*SC embedded in staff induction programs*	Participant 3	*App development similar to COVID with engagement*	Participant 3
*Nonjudgmental service and empowering*	Participant 11	*Training staff*	Participant 3	*Psychologist/counselor*	Participants 3, 15
*Consent from the patient and develop* plan	Participants 9, 16	*Reinforce SC message at all interactions*	Participants 17, 10	*SC embedded in cancer services*	Participants 12, 14
*Use hospital—patients are attending*	Participants 1, 4, 11, 12	*Link to community services*	Participant 3	*NRT prescription*	Participants 3, 8, 11
*Longer-term follow-up (can include GP and community referrals)*	Participants 1, 3	*Documenting every conversation*	Participant 10	*Use and extending Daffodill nurses*	Participant 9
*Face-to-face service preferred*	Participants 4, 11, 16	*Appointment system in hospital with follow-up 24–48 h after referral by SC facilitator*	Participant 4	*Nicotine spray availability*	Participant 9
*Online session is better than phone follow-up*	Participant 11	*Interpreters available*	Participant 11	*Family support to quit*	Participant 10
*Discuss SC early—but not at diagnosis*	Participants 2, 9, 14, 10			*More staff employed*	Participant 17
*Discuss during treatment*	Participant 12			*Increased nurse prescribing to include NRT medications*	Participant 8
*Discuss years after diagnosis*	Participant 11				
*Discuss after treatment*	Participants 11, 13				
*Discuss relapsing*	Participants 9, 11				

^1^Patient-focused solutions to support smoking cessation—highlighted by specific participants during interviews.

^2^Solutions that hospitals could adopt to support smoking cessation—highlighted by specific participants during interviews.

^3^Wider systems-level/national solutions for smoking cessation—highlighted by specific participants during interviews.

I think it is great and there is really practical information, but you also have to have, like not everybody in this country can read English, not everyone is tech savvy, not everyone can read. And I know at least when we link somebody of a different nationality with our facilitator, we can book an interpreter at the same time and that is crucial (HCP 11, Nurse, 13 years’ experience).

I think it is everyone’s role, all healthcare professionals, it doesn’t just come down to the smoking cessation nurse or people doing quit.ie, it is every healthcare professional’s role to do it (HCP 14, Nurse, 6 months experience).

Recommendations from participants in Table 2 present improvements in the SC services to be considered at patient, HCP, and organizational levels.

## Discussion

**Figure 1.**
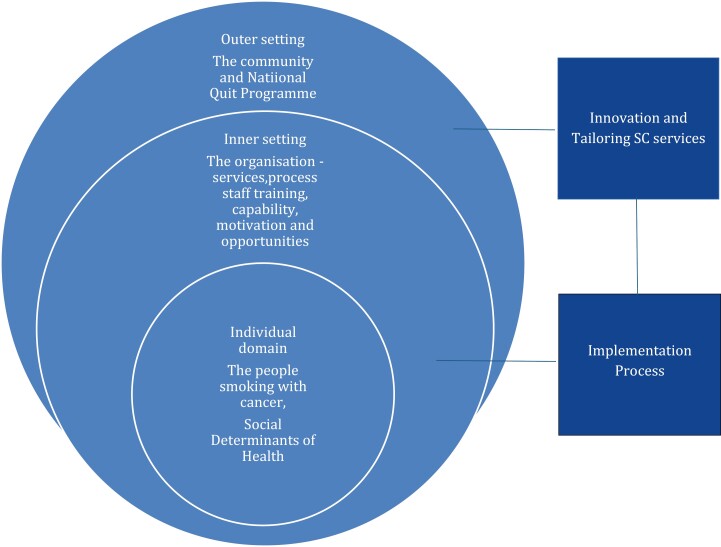
Tailoring solutions for smoking cessation (SC) in cancer services.

This Irish study of HCPs working in specialist cancer services, sharing their experiences of engaging in SC practices, demonstrates therapeutic relationship-building practices, highlights unmet needs, and presents organizational systems-level solutions (Supplementary File 2; Table 2 and Figure 1). This study represents the unique engagement of patient and public involvement in the research design and methods adopted. The findings presented in this study are consistent with recent international evidence, which suggests that professional nihilism exists and that SC practices in the United States vary despite developments and investments.^8,10,21^ Developing and nurturing the therapeutic relationship are fundamental, and findings from this study identify structural and personal barriers that impact engagement. The MECC program^22^ is a lauded process of engagement to *Ask, Advise, and Act* to support SC activities. Evidence in this study demonstrates its use alone may be inadequate, as it does not foreground the complexities for those who smoke and have a cancer diagnosis, especially if patients do not *demonstrate their eagerness for SC* when dealing with a diagnosis and the effects of therapeutic interventions. Engagement with MECC is not a mandatory requirement for employment or data captured on who attends.^11^ Patients are in an unfamiliar context with reduced capabilities, opportunities, or motivation, impacting their behaviors and choices.^23^ Evidence emerging in this study is consistent with recent international and longstanding research demonstrating the challenges for HCPs and patients^5–7,24^; and despite all of the evidence, smoking remains a sensitive topic. Patients do expect to be asked about their smoking history and will engage if it’s with “*the right person.*”^5(p.7)^

In this study, several participants were aware of the SC services offered in their organization but articulated inconsistencies in systems and processes in obtaining SC histories. There was a lack of familiarity with who was responsible for engaging or initiating SC conversations. Structural barriers with limited time during clinics intensify workload challenges. An absence of electronic systems for recording SC information, embedding SC question templates into hospital documentation, or automatic referral to SC practitioners adds to the burden on the clinical workforce. A recent review of clinical notes from a US oncology center suggests inequity in documenting and discussing smoking histories for all patients and variations for those with prostate cancer patients when compared with head and neck, lung, or breast cancer patients.^25^ Relying on HCPs working within complex care clinical departments diminishes SC to a secondary conversation and, for some, a question that may be asked only once during multiple clinical interactions, including diagnosis and treatment or may involve a referral to a passive system where the patient is required to make the first connection to SC services. Embedding SC training in cancer training programs should be mandatory in addition to MECC training and supporting the development of opt-out SC programs.^9,26^ These factors help HCPs’ knowledge and assist in overcoming personal beliefs, understanding their fears of exacerbating patients’ guilt and identifying solutions to enable conversations supported by international evidence.^5,9,21,25,26^

Cancer services are complex, and how best to tailor SC service may be informed using complexity theory frameworks.^27^Table 2 and Figure 1 demonstrate the many systems and contexts that must be considered for tailoring the solutions that emerged from the findings in this study. Suggested systems-level approaches include improved communication, use of innovation and digital solutions, including social media, and SC app development. Meso or organizational-level approaches focus on embedding SC within cancer services, such as developing an active referral system and pathway to document smoking histories. Current referral policies and expectations are based on the capability and motivation of patients. HCPs’ attitudes and beliefs may be misplaced on what is feasible in the absence of discussing and codeveloping a plan with patients (individual domain) within an active referral system, which is critical.

The current study was completed during a consultation period and publication of National Tobacco Control Clinical Guidelines.^28^ Irish national clinical guidelines support a consistent approach for routine SC with the population, and this includes the availability of free NRT from SC services, and provision of one-to-one and group consultations, but they do not specifically guide practitioners in populations in complex settings or with cancer. Our suggestions to improve SC practices may require policy changes locally, including expanding the registered nurse prescribers’ formulary to include prescriptive authority for NRT medications. There is an appetite for change, given the development of registered nurse prescribers^29^ and current international and national discourse.^30–32^

Integration with community providers and GPs is necessary.^10,33^ However, a recent survey of GPs in Ireland highlights discrepancies in their knowledge base and a need for more training on SC.^34^ Lyons et al.^11^ reported a lack of SC services and systems in a recent Irish audit of cancer centers, including a lack of on-site access 24/7 to NRT. The use of artificial intelligence to develop SC interventions may provide further options where appropriate.^35^ Findings in this study are consistent with international research demonstrating the suboptimal SC services for patients with cancer and those with respiratory disease and peripheral artery disease,^21,36^ thus emphasizing a need for codeveloped and tailored pathways for those who have the most to gain by quitting smoking and improving their health outcomes and using a systems lens to develop solutions.^37,38^

### Limitations

Due to the qualitative nature of this study, the findings may not be representative of the experiences and views of all HCPs working within specialist cancer centers. It may be a result of recruitment procedures that others could not participate in due to competing challenges in clinical practice and a COVID-19 context. The recruitment procedures provided an opportunity for all working in each of the national centers to participate. Due to COVID-19, the study team was not permitted access to hospitals, or interview participants in person. It is recognized that other HCPs, in more comprehensive clinical settings (not specialist cancer centers), would benefit from being included in future studies. However, importantly, this study provides valuable insight into the experiences of HCPs working in specialist centers, and findings can be used to inform the development of SC services for patients with cancer.

## Conclusions

This study confirms the unmet needs of cancer patients regarding SC services and argues for reevaluating the *status quo*, to consider systems-level solutions and continuing training and upskilling requirements of HCPs in oncology.

## Supplementary Material

Supplementary material is available at *Nicotine and Tobacco Research* online.

ntae185_suppl_Supplementary_Material_S1

ntae185_suppl_Supplementary_Material_S2

## Data Availability

Data are not publicly available due to ethical approval conditions in 2021.
