# Supplementary material for: A Qualitative Study to Understand the Barriers and Facilitators in Smoking Cessation Practices Among Oncology Health Care Practitioners in One Health System
Source: Nicotine Tob Res. 2024 Jul 22;27(2):199–207. doi: 10.1093/ntr/ntae185 (PMC11750740; doi:10.1093/ntr/ntae185)
Supplement: ntae185_suppl_Supplementary_Material_S1 [file ntae185_suppl_supplementary_material_s1.docx]

**Irish Cancer Society Funded Smoking Cessation for Cancer Patients in Ireland: A Scoping and Feasibility Initiative**

Demographics section for semi-structured interviews with oncology health professionals and allied healthcare professionals (recorded with consent)

| Professional Role Please tick relevant box | | |
| --- | --- | --- |
| Registered Nurse | **Medical Doctor** | **Allied Healthcare Professional** |
| Staff Nurse 🞏 | Intern **🞏** | Health Promotion Officer **🞏** |
| Oncology Nurse*🞏 | Senior House Officer **🞏** | Smoking Cessation Officer **🞏** |
| Breast Care Nurse 🞏 | Registrar **🞏** | Radiation Therapist **🞏** |
| Clinical Nurse Specialist 🞏 | Specialist Registrar (Oncology) **🞏** | Speech and Language Therapist **🞏** |
| Clinical Nurse Manager 🞏 | Consultant Medical Oncologist **🞏** | Physiotherapist **🞏** |
| Advanced Nurse Practitioner 🞏 | Consultant Surgical Oncologist **🞏** | Medical Social Worker **🞏** |
| Clinical Trials Nurse 🞏 | Consultant Radiation Oncologist **🞏** | Dietician **🞏**  Occupational Therapist **🞏** |
| Irish Cancer Society Daffodil Centre Nurse 🞏 |  |  |
| Other (please specify) | | |

*Staff Nurse with postgraduate qualification in Cancer Nursing

| Gender (Please tick relevant box) | |
| --- | --- |
| Male🞏 |  |
| Female🞏 |  |
| Nonbinary🞏 |  |
| Prefer not to say🞏 |  |

| Primary Work Setting (Please tick relevant box(s)) | | | | | |
| --- | --- | --- | --- | --- | --- |
| Medical Oncology | | **Surgical Oncology** | | **Radiation Oncology** | |
| Inpatient Ward🞏 |  | Inpatient Ward**🞏** |  | Inpatient Ward**🞏** |  |
| Outpatients  Clinic🞏 |  | Outpatient Clinic**🞏** |  | Outpatient Clinic**🞏** |  |
| Outpatients  (Oncology/Haematology Day Ward)🞏 |  | Rapid Access Clinic**🞏** |  | Radiotherapy Treatment Unit**🞏** |  |
| ICSDaffodil Centre🞏 |  |  |  |  |  |
| Clinical Trials Unit🞏 |  |  |  |  |  |
| Other🞏 Specify |  |  |  |  |  |

How long are you working in current professional role? Duration (years in current role) ___________

Do you smoke?

Current smoker **🞏**Ex Smoker**🞏** Never smoked **🞏**

**Do you routinely ask your patients if they smoke?**

**Y 🞏 N 🞏 If answer is YES - go to Section 2**

**If answer is NO – go to Section 3**

**SECTION 2**

**I’d like to ask some questions about how you discuss smoking cessation with patients…….**

|  |
| --- |
| …. When you talk to your patients about quitting is there a particular approach you use? |
|  |
| At what point/ clinic visit for example would you talk about quitting smoking (*pre diagnosis, diagnosis, during treatment, post treatment, admission for complications, follow ups, other)* |
|  |
| Do you routinely speak to patients about the impact of smoking on treatment outcomes and/or risk of recurrence/second primary cancer… can you talk to me about this? |
|  |
| In your experience is there a ‘best time’ to have this conversation with a patient? *(at diagnosis, during chemo, during follow up at OPD, GP in community, much later after treatment fully finished etc)?* |
|  |
| If a family member was present would you include them in the discussions with the patient about quitting smoking? *If yes,* can you talk to me about your experience of this? |
|  |
| Do you talk about any smoking cessation interventions with patients? Can you tell me a little bit more about that? |
|  |
| What (if anything) do you recommend/prescribe? |
|  |
| What (if any) smoking cessation services do you refer patients to? *(for example HSE Quit line, GP, Hospital service if available, community)* |
|  |
| In your view what are the facilitators to discussing smoking cessation with patients and family members? |
|  |
| In your view are there any challenges discussing smoking cessation with patients and families? |
|  |
| Can I ask if there has been any change in your approach to advising your patients about smoking cessation as a result of COVID-19? |

**I’d now like to ask you some questions that will help us in developing a smoking cessation service for people who have cancer.**

|  |
| --- |
| *How do you feel about talking about smoking with patients who have cancer? Can you talk to me about this?* |
|  |
| Do you think discussing smoking cessation is part of your role?  *If yes,* can you explain why your role is important in discussing smoking cessation?  *If NO,* can you talk to me about that …. who you think should discuss smoking cessation and supports with patients and why? |
|  |
| In your view, what are features of an effective smoking cessation intervention for patients who have cancer? C*an you describe what an ideal service would look like?*  (*with reference to feasibility, acceptability, likelihood of uptake of smoking cessation supports/services*) |
| Can I just check - do you routinely ask your patients if they vape / use e-cigarettes?  Y 🞏 N 🞏 |
|  |
| Is there anything else you would like to add? |
|  |

**Thank you …………**

**Section 3**

**You said you don’t discuss smoking with patients, I’d like to ask some questions about this ……**

|  |
| --- |
| Was there ever a time/period you did ask a patient about quitting smoking?  if yes,  Can you tell me a little more about this ….. was there an event or reason that prompted you to discontinue the conversations…... |
|  |
| I appreciate you don’t routinely talk to patients about quitting smoking, can I ask have you ever spoken to family members…….if yes,. can you talk to me about this ? |
|  |
| In your view what are the challenges to discussing smoking cessation with patients and family members? |
|  |

**I’d now like to ask you some questions that will help us in developing a smoking cessation service for people who have cancer.**

|  |
| --- |
| How do you feel about talking about smoking with patients who have cancer? Can you talk to me about this? |
|  |
| Do you think discussing smoking cessation is part of your professional role?  *If yes,* can you explain why your role is important in discussing smoking cessation?  *If NO*, can you talk to me about that? Who you think should discuss smoking cessation and supports with patients and why? |
|  |
| In your view, what are features of an effective smoking cessation intervention for patients who have cancer? Can you describe what an ideal service would look like?*(with reference to feasibility, acceptability, likelihood of uptake of smoking cessation supports/services)* |
| In your opinion is there a best time to a conversation with a cancer patient about quitting smoking? |
| Can I just check - do you routinely ask your patients if they vape / use e-cigarettes?  Y 🞏 N 🞏 |
|  |
| Is there anything else you would like to add? |

Thank you
