# Supplementary material for: A Qualitative Study to Understand the Barriers and Facilitators in Smoking Cessation Practices Among Oncology Health Care Practitioners in One Health System
Source: Nicotine Tob Res. 2024 Jul 22;27(2):199–207. doi: 10.1093/ntr/ntae185 (PMC11750740; doi:10.1093/ntr/ntae185)
Supplement: ntae185_suppl_Supplementary_Material_S2 [file ntae185_suppl_supplementary_material_s2.docx]

|  | Quotations supporting themes | | |
| --- | --- | --- | --- |
|  | Themes | Subthemes | Examples |
| 1 | Timing and Knowledge | Workload | *Probably one of the biggest things would be time constraints. The other thing would be the appropriateness of the conversation* (HCP 16, Doctor, 2 years’ experience) |
|  |  | *Competing Demands* | *I actually sometimes question whether when they are on treatment is the actual best time. I would sometimes think maybe it is not because, as I said, by the time they come to us they have already started treatment, some of them have already had surgery, some of them have had chemotherapy. And it may be late, I would feel it is possibly a little bit late to address it with them. It might be better that it be addressed prior to them coming to us and then being reinforced by us………* *And then on top of it you are addressing maybe this with them for the first time. They can, by the time they get to us be a bit overwhelmed to begin with and I think as well for a lot of people with addictions the stress of having a cancer diagnosis, their way of coping with stress may be to turn to alcohol or something like smoking and therefore it is actually a coping mechanism for them.* (HCP 10,Allied HCP, 14 years’ experience) |
|  |  | *Understanding of services* | *Now, or the Irish Cancer Society in fairness for women it's offered for free, I think, for men I'm not sure if it's offered for free now would it wasn't, and I would ask them to link into the quick plan on the HSE website* (HCP 3, Doctor, 10 years’ experience) |
|  |  |  | *… actually sent a referral in for them but I don't know, when they are inpatients I don't know actually what they do… I really don't know. I know I attended a class previously when I was in the oncology time and I know a few girls came and gave classes about the smoking cessation programme and all those things. Since then I never attended a programme. I always refer people to preventative medicine medicine, and I don't know since then what happens.* (HCP 17, Nurse, 3.5 years’ experience) |
| 2 | Building a Relationship | *Person centred care* | *Being understanding that if somebody isn't in a place, even a couple of years after a cancer diagnosis that you just continue to bring it up and continue to offer them supports. Maybe they weren't aware of certain things that were available at the time they were diagnosed but now there is more supports and maybe they would like to try now* (HCP 11, Nurse,, 13 years’ experience) |
| 3 | Frequent asking and infrequent action | *Signposting* | *I do direct them to the HSE, there is an HSE smoking cessation web page and there is quit.ie. I know there is community services and things as well if they were interested in that. But I usually start with the smoking cessation nurse. If it was something that they were interested in we have the Irish Cancer Society just down the hall so I can get them a patient information leaflet from there as well and then direct them to the website. But that is on the patient information leaflet as well* (HCP 4, Nurse , 4 years’ experience ) |
|  |  | *Advising* | *We always advise them, if they are current smokers, we always advise them about smoking cessation. We always tell them whatever they can do, like if they want to try patches or gum or things like that we can give prescription. Or if you want …. we can send a referral. Some of the patients are happy to send a referral, some of the patients are happy to take the patches. Some of them will think about it and come back to us* (HCP 17, Nurse, 3.5 years’ experience |
|  |  | *Action* | *… generally would link them up to, let them know what services are available so the smoking cessation officer in the hospital. That is generally what, or any of the quit apps or quit.ie or things like that. To be perfectly honest with you a lot of the time I would and I would broach it with them and I would talk to them about it but I wouldn't delve into it too much. I discuss it, I mention it but I wouldn't have a comprehensive discussion with them. I would just say what is out there and then I' would leave it* (HCP 13, Nurse, 13 years’ experience) |
|  |  |  | *I offer the help of our smoking cessation nurse and we have a referral system on our central system and she is really, really good, she gets into contact with them really quickly and is really accommodating with appointments.* (HCP 4, Nurse, 4 years’ experience). |
|  |  | *Inaction* | *….you have definitely opened up my mind again to bring it back to conversation more with patients and especially the family members, that is a huge point because that is a massive deterrent with people don't give up is the family members, if they continue to smoke (HCP 13, Nurse, 17 years’ experience)* |
| 4 | Removing barriers and tailoring a system | *Change the system* | *I think the idea is that if we were to take a more structured approach to patient assessment, recommendations on treatments and including smoking cessation, one of the more prominent element is the kind of prevention of long term harm, that may be the way to do it.* (HCP 1, Doctor , 15 years’ experience ) |
|  |  | *What we can do* | *I think the quit website is really good and the information is very good but actually having that support one on one, I think the rate of cessation would be higher and the willingness to give up would be higher. I know the lady that we have is just fabulous with our patients and she is very down to earth, very non-judgemental and exactly what people need at this time in their lives. So I think that is crucial, having a designated smoking cessation department or facilitator* (HCP 11, Nurse, 13 years’ experience) |
|  |  |  | *I need to develop myself, I think it is out of killing them with kindness slightly* (HCP 13, Nurse, 17 years’ experience) |
